# Utility of Polymerase Chain Reaction in Nasopharyngeal Swabs for Identifying Respiratory Bacteria Causing Community-Acquired Pneumonia

**DOI:** 10.1128/spectrum.00379-22

**Published:** 2022-05-18

**Authors:** Yoris Demars, Thomas Brahier, David C. Rotzinger, René Brouillet, Katia Jaton, Onya Opota, Noémie Boillat-Blanco

**Affiliations:** a Infectious Diseases Service, University Hospital and University of Lausanne, Lausanne, Switzerland; b Institute of Microbiology, University Hospital and University of Lausanne, Lausanne, Switzerland; c Department of Diagnostic and Interventional Radiology, University Hospital and University of Lausanne, Lausanne, Switzerland; Montefiore Medical Center and Albert Einstein College of Medicine

**Keywords:** FilmArray, *Haemophilus influenzae*, *Moraxella catarrhalis*, NP, PCR, POCT, *Streptococcus pneumoniae*, diagnostics, molecular diagnostics, pneumonia

## Abstract

Timely identification of a pathogen in lower respiratory tract infections (LRTI) can support appropriate antibiotics use. The difficulty of obtaining lower respiratory tract (LRT) samples limits the utility of point-of-care syndromic molecular assays. We assessed the performance of the FilmArray Pneumonia plus panel (FilmArray PP) in nasopharyngeal (NP) swab for detection of Streptococcus pneumoniae, Haemophilus influenzae, and Moraxella catarrhalis. Patients in the study included retrospectively consenting adults who attended the emergency department of Lausanne University Hospital between February 2019 and August 2020 for a community-acquired LRTI, with available NP swab and a high-quality LRT sample. These samples were tested with the FilmArray PP (cutoff of ≥10^4^ copies/mL). Positive (PPA) and negative percent agreement (NPA) of FilmArray PP in NP swab were calculated, using (i) FilmArray PP in LRT sample and (ii) standard microbiological tests as reference standards. To assess the performance of a lower detection cutoff, NP samples were also tested with an in-house PCR (cutoff of ≥10 copies/mL) for S. pneumoniae and H. influenzae. Overall, 118 patients were included. FilmArray PP in LRT sample and standard microbiology tests detected S. pneumoniae in 19/118 and 12/118, H. influenzae in 44/118 and 19/118, and M. catarrhalis in 14/118 and 0/118, respectively. Using LRT FilmArray PP as reference, PPA and NPA of FilmArray PP on NP were 58% and 100% for S. pneumoniae, 61% and 100% for H. influenzae, and 57% and 99% for M. catarrhalis. Using standard diagnostic tests as reference, PPA and NPA were 58% and 96% for S. pneumoniae, 74% and 87% for H. influenzae, and indefinite and 92% for M. catarrhalis. Using a lower cutoff on NP (≥10^2^ copies/mL), PPA was 68% for S. pneumoniae and 77% for H. influenzae with LRT FilmArray PP as reference. FilmArray PP in NP swabs has a limited PPA for identifying the most common etiologies of community-acquired LRTI irrespective of the reference standard, preventing its use for withholding antibiotics. The PCR detection cutoff does not explain the low PPA. The excellent NPA suggests the use of NP PCR results for rapidly targeted antimicrobial therapy.

**IMPORTANCE** Timely identification of a pathogen in patients with lower respiratory tract infections is of paramount importance to avoid inappropriate antibiotic prescription. We aimed to evaluate the performance of a rapid syndromic molecular assay in nasopharyngeal swabs for identifying the most common bacterial causes of lower respiratory tract infections in adults (Streptococcus pneumoniae, Haemophilus influenzae, and Moraxella catarrhalis). Our data show that nasopharyngeal molecular assay has a good concordance with lower respiratory tract sample when positive but not when negative. A positive result is therefore concordant with a lower respiratory tract infection and can be used to target antibiotics. Nevertheless, a negative result does not have a good concordance, so it cannot be used to withhold antibiotics. Our findings illustrate the potential utility of these easily collected samples for the management of patients with lower respiratory tract infections.

## INTRODUCTION

Lower respiratory tract infections (LRTIs) are one of the most common motives to attend emergency departments (ED) and the main reason for inappropriate antibiotic prescription (1). Timely identification of a pathogen in patients with LRTIs may support optimization of antibiotics use.

However, current microbiological methods identify a pathogen in only 40 to 60% of patients with LRTI (2–4) and rely mainly on culture, which is a slow process (5). Rapid syndromic molecular assays targeting respiratory bacteria and viruses are now available (6). Their use in lower respiratory tract (LRT) samples increases pathogen detection to 83 to 87% of patients with LRTI (7, 8), suggesting their utility to deescalate or discontinue antibiotics (7–11). However, less than 50% of the patients produce sputa (12), limiting the impact of using these rapid syndromic molecular assays. Nasopharyngeal (NP) swabs are easy to obtain in all patients and may replace LRT samples, as it has been supposed that LRTIs could be provoked by an organism in the nasopharynx that spread in the lower tract (13). Although molecular tests in NP swabs are accepted for viruses and atypical bacteria such as Mycoplasma pneumoniae and Chlamydia pneumoniae (14), few studies evaluated their performance on upper respiratory tract samples (URT) for the most common bacterial causes of community-acquired LRTIs, such as Streptococcus pneumoniae, Haemophilus influenzae, and Moraxella catarrhalis. Four studies evaluated identification of S. pneumoniae by real-time PCR (RT-PCR) in NP specimen of patients with community-acquired pneumonia (CAP) with negative percent agreements (NPA) of >90% but with wide differences in positive percent agreement (PPA; 35% to 82%) using standard microbiological tests as gold standard (15–18). No study evaluated the validity of PCR in the NP swabs for other bacteria. To our knowledge, only two studies assessed the performance of a syndromic molecular assay including community-acquired bacteria on URT samples (NP and oropharyngeal [OP] swabs), and they gave contradictory results, as one concluded that sputum was more reliable than NP/OP (19), while the other concluded that sputum had no advantage to detect a pathogen over URT samples (20).

Our study aims to define the performance of the FilmArray Pneumonia plus panel (FilmArray PP) in NP swabs for identifying the most common bacterial causes of LRTIs (S. pneumoniae, H. influenzae, M. catarrhalis) in adults using (i) FilmArray PP in LRT sample and (ii) standard microbiological tests as reference standards. Our study also aims to test the performance of different PCR threshold cutoffs on NP swabs.

## RESULTS

**FIG 1:**
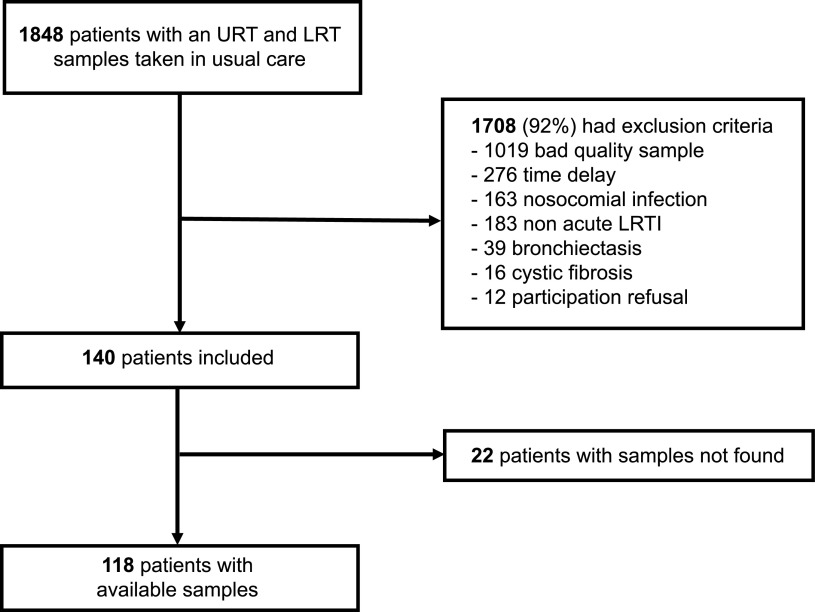
Flowchart of study participants. URT, upper respiratory tract; LRT, lower respiratory tract; LRTI, lower respiratory tract infection.

Among 1,848 patients (350 during phase 1 and 1,498 during phase 2) eligible with NP swab and LRT samples available, 1,708 (92%) were excluded (1,019 had a poor-quality LRT sample, 276 a sample collected more than 48 h from admission or with ≥24 h between NP swab and LRT sample, 163 were hospitalized in an acute care hospital in the last 3 months, 183 did not have an acute LRTI, 39 had bronchiectasis, 16 had cystic fibrosis, and 12 refused to participate). After excluding 22 patients because we did not find their samples, our study population consisted of 118 patients (27 from phase 1 and 91 from phase 2) (Fig. 1).

### Patient characteristics.

Median age was 71 years (interquartile range [IQR], 56 to 78), 43 (36%) patients were female, and 34 (29%) had chronic obstructive pulmonary disease (COPD) (Table 1). Overall, 88 (75%) had an infiltrate on chest X-ray or CT scan. A total of 108 (96%) patients received an antibiotic, which was started before collecting the LRT sample in 55 (47%) patients. A total of 97 (87%) patients were admitted to the hospital, 13 (11%) were admitted to the ICU, and 4 (3.3%) died.

**TABLE 1:** Characteristics of study participants^*a*^^,^^*g*^

Characteristic	No. of patients (*n* = 118)
Demographics	
Female sex, *n* (%)	43 (36)
Age (yrs), median (IQR)	71 (56, 78)
Residence in nursing home, *n* (%)	8 (6.8)
Current smoker, *n* (%)	36 (31)
Alcohol misuse, *n* (%)	18 (15)
Coexisting disorder, *n* (%)	
Any	102 (86)
Chronic obstructive pulmonary disease	34 (29)
Heart failure	24 (20)
Active neoplasia	23 (20)
Diabetes	20 (17)
Asthma	18 (15)
Neurological disorder^*b*^	17 (14)
Chronic renal failure^*c*^	17 (14)
Immunosuppressive treatment	16 (14)
Other^*d*^	10 (8.4)
Symptoms, *n* (%)	
Expectoration	82 (85)
Dyspnea	78 (76)
History of fever	66 (64)
Thoracic pain	20 (24)
Signs in emergency department	
Systolic blood pressure (mm Hg), median (IQR)	124 (110, 137)
Diastolic blood pressure (mm Hg), median (IQR)	72 (64, 81)
Heart rate (bpm), median (IQR)	90 (80, 100)
Respiratory rate (vpm), median (IQR)	21 (18, 25)
Oxygen saturation (%), median (IQR)	94 (92, 96)
Temp (°C), median (IQR)	37 (37, 38)
Abnormal lung auscultation, *n* (%)	82 (75)
Severity score, *n* (%)	
CURB-65 ≥ 2	47 (50)
qSOFA ≥ 2	17 (17)
Laboratory findings at first consultation	
Leukocyte count (g/L), median (IQR)	11 (8, 15)
CRP (mg/L), median (IQR)	74 (29, 154)
Creatinine (μmol/L), median (IQR)	86 (71, 122)
Radiology, *n* (%)	
Infiltrate on chest imaging^*e*^	88 (75)
Antibiotics administration, *n* (%)	
Antibiotics administrated	108 (96)
Antibiotics before LRT sample collection^*f*^	55 (47)
Clinical outcome, *n* (%)	
Hospital admission	97 (87)
Intensive care unit admission	13 (11)
Intubation	10 (8)
Death	4 (3.3)

a IQR, interquartile range; BP, blood pressure; bpm, beat per minute; vpm, ventilation per minute; CRP, C-reactive protein; LRT, lower respiratory tract.

b Cerebrovascular disease, dementia, Parkinson.

c KDIGO stage ≥ G3a.

d Terminal renal failure or dialysis (*n* = 2), hepatic failure (*n* = 3), HIV infection (*n* = 5).

e All participants had a chest imaging performed (29% a CT scan, 71% only a radiography).

f 12/55 were started >48 h before sample collection.

g Missing values: smoking status 1, CURB-65 23, qSOFA 19, fever 14, expectoration 21, dyspnea 15, thoracic pain 33, auscultation status 9, respiratory rate 19, oxygen saturation 5, fraction of inspired oxygen 3, leukocytes 5, CRP 2, urea 10, creatinine 2.

### Microbiological documentation.

Based on the standard composite microbiology diagnostic tests, 56% (66/118) of patients had a documented infection, while 44% (52/118) did not have any detected pathogen. A total of 39% (46/118) had a bacterium detected, 24% (28/118) a virus, and 6.8% (8/118) a virus and a bacterium. The most frequent pathogens were H. influenzae (16%), S. pneumoniae (10%), and influenza virus (10%) (see Table S1 in the supplemental material).

Based on the FilmArray PP in the LRT samples (112 sputa, 4 lung aspirates, 2 bronchoalveolar lavages), 88% (104/118) of patients had a documented pathogen, while 12% (14/118) did not. A total of 76% (90/118) patients had a bacterium detected, 57% (67/118) a virus, and 45% (53/118) a virus and a bacterium. The most frequent pathogens were H. influenzae (37%), S. aureus (25%), S. pneumoniae (16%), M. catarrhalis (12%), enterovirus (25%), and influenza virus (14%) (Table S1).

Based on the FilmArray PP in the NP swab, 82% (97/118) of patients had a documented pathogen, while 18% (21/118) did not. A total of 62% (73/118) of patients had a bacterium detected, 48% (57/118) a virus, and 13% (15/118) a virus and a bacterium. The most frequent pathogens are similar to those identified by FilmArray PP in the LRT samples (Table S1).

Compared to standard microbiology, the FilmArray PP increased the diagnostic yield of bacteria by 95% (from 39% using standard microbiology to 76% using FilmArray PP in LRT samples).

### Identification of the three bacteria of interest: S. pneumoniae, H. influenzae, and M. catarrhalis.

**FIG 2:**
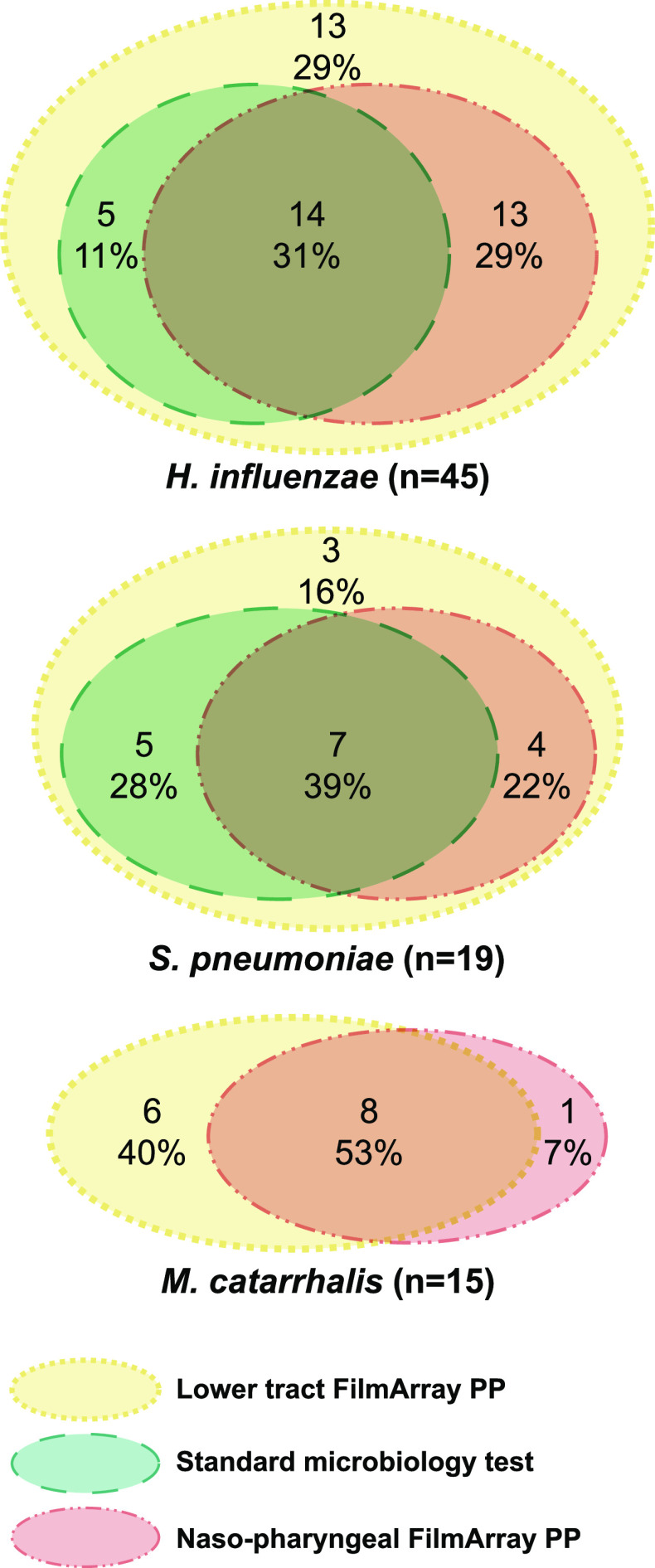
Venn diagrams showing the concordance between the three microbiological tests (FilmArray Pneumonia Panel plus in nasopharyngeal swab and in lower respiratory tract sample, and the composite standard microbiological tests) for Streptococcus pneumoniae, Haemophilus influenzae, and Moraxella catarrhalis in the study population (*n* = 118).

Standard microbiology and FilmArray PP in LRT samples and in NP swabs identified S. pneumoniae in 12 (10%), 19 (16%), and 11 (9.3%), H. influenzae in 19 (16%), 44 (37%), and 27 (23%), and M. catarrhalis in none, 14 (12%), and 9 (7.6%) patients, respectively. Figure 2 shows the concordance of the three diagnostics tests for S. pneumoniae, H. influenzae, and M. catarrhalis. Using FilmArray PP in LRT sample, the performance for detecting the three bacteria was similar using a positive cutoff of >10^4^ or >10^5^ copies/mL. Concordance between standard and molecular diagnostic tests in LRT samples was high for S. pneumoniae (94% of overall agreement) and lower for H. influenzae (79%) and M. catarrhalis (88%).

### Performance of the FilmArray in NP swabs of adults with community-acquired lower respiratory tract infection.

Table 2 shows the performance of the FilmArray PP in NP swabs for the detection of S. pneumoniae, H. influenzae, and M. catarrhalis in patients with community-acquired LRTI. We calculated the performance according to two reference standards. Using FilmArray PP on LRT sample as the reference standard, the PPA, NPA, positive predictive values (PPV), and negative predictive values (NPV) were, respectively, 58%, 100%, 100%, and 93% for S. pneumoniae, 61%, 100%, 100%, and 81% for H. influenzae, and 57%, 99%, 89%, and 94% for M. catarrhalis. Using the standard microbiology results as reference standard, the PPA, NPA, PPV, and NPV were, respectively, 58%, 96%, 64%, and 95% for S. pneumoniae, 74%, 87%, 52%, and 95% for H. influenzae, and undefinable, 92%, 0%, and 100% for M. catarrhalis.

**TABLE 2:** Performance of FilmArray Pneumonia Panel plus (FilmArray PP) in nasopharyngeal (NP) swabs for detection of S. pneumoniae, H. influenzae, and M. catarrhalis in adults with community-acquired respiratory tract infections (*n* = 118 patients) compared to various reference standards: FilmArray PP on a good-quality lower respiratory tract (LRT) sample with a positive cutoff value of >10^4^ copies/mL or with the standard microbiology results (lower respiratory tract sample culture, hemocultures, and S. pneumoniae urinary antigen)^*a*^

Target organism	Reference standard	No. of positive samples		PPA	NPA	OPA	LR+	LR−	PPV	NPV
		NP	Reference standard							
S. pneumoniae	LRT PCR FilmArray PP	11	19	0.58	1	0.93	Infinite	0.42	1	0.93
	Standard microbiology	11	12	0.58	0.96	0.92	15.46	0.43	0.64	0.95
H. influenzae	LRT PCR FilmArray PP	27	44	0.61	1	0.86	Infinite	0.39	1	0.81
	Standard microbiology	27	19	0.74	0.87	0.85	5.61	0.3	0.52	0.95
M. catarrhalis	LRT PCR FilmArray PP	9	14	0.57	0.99	0.94	59.43	0.43	0.89	0.94
	Standard microbiology	9	0		0.92	0.92			0	1

a Positive percent agreement (PPA), negative percent agreement (NPA), overall percent agreement (OPA), positive likelihood ratio (LR+), negative likelihood ratio (LR−), positive predictive value (PPV), negative predictive value (NPV).

Considering only patients with a respiratory sample taken before antibiotics administration (*n* = 55), the NPA of FilmArray PP on NP sample compared to the standard microbiology results as reference standard was not different (57%, 85%, and undefinable for S. pneumoniae, H. influenzae, and M. catarrhalis, respectively).

### Performance of the FilmArray in NP swabs using lower cutoff of detection.

Hypothesizing that the performance of the PCR could be better with a lower positive cutoff, we performed an in-house PCR for S. pneumoniae and H. influenzae in all respiratory samples. Considering a cutoff value of ≥10^2^ copies per mL on the NP swab, the PPA was slightly higher than a cutoff of ≥10^4^ copies per mL using FilmArray PP on LRT samples as the reference standard: 58% to 68% for S. pneumoniae and 61% to 77% for H. influenzae (Table S2). The performance was similar to that with standard microbiology as the reference standard but also to that of in-house PCR on LRT samples.

## DISCUSSION

The difficulty in obtaining high-quality sputum and the long time-to-result of standard “culture-based” microbiological analyses are major limitations for timely identification of a pathogen in patients with LRTI and tailored use of antibiotics. Our data show that molecular analyses in NP swabs for identifying the most common bacterial causes of pneumonia have a high NPA and a limited PPA, suggesting their utility to promptly use targeted antibiotics in case of positive results but not their utility to withhold antibiotics in case of negative results. Lowering the detection cutoff of the molecular analysis in NP swab only slightly improves the PPA.

Few studies assessed the performance of molecular diagnostic tests on URT samples to identify the most common causes of bacterial community-acquired pneumonia. Comparison between studies is difficult, as they use different URT samples (NP, oropharyngeal [OP], or combined samples), different PCR targets, and different reference standards (PCR on LRT samples or standard microbiological tests).

We found a limited PPA of molecular analyses in NP swabs, which is concordant with the majority of previous studies. Two studies compared the performance of a syndromic molecular assay (TaqMan Array Card Technology) on a combination of URT samples (NP and OP) using the same molecular assay on high-quality sputum as reference standard in hospitalized patients with CAP: the first one, performed in the United States, found a PPA between 50 and 80% for S. pneumoniae, H. influenzae, and M. catarrhalis (19), and the second one, conducted in Kenya (39% HIV-infected patients), found a PPA between 72 and 80% for S. pneumoniae, H. influenzae, and M. catarrhalis (20). Three studies evaluated the performance of *lytA* RT-PCR in NP specimens for S. pneumoniae diagnosis using standard microbiology as reference standard in patients with CAP. In line with our study, two studies performed in Western Europe found a PPA between 44 and 72% (17, 18). However, the third study, performed in South Africa among HIV-infected adults, showed a higher PPA at 82% (15). This may be explained by a higher bacterial load in this immunocompromised population with CAP.

The excellent NPA of molecular analyses in NP swabs found in our data is in line with all previously performed studies. Using a syndromic molecular assay (TaqMan Array Card Technology) on a URT sample and high-quality sputum in hospitalized patients with CAP, a study performed in the United States and a study conducted in Kenya confirmed the high NPA (>98% and 88 to 93%, respectively) (19, 20). Using *lytA* RT-PCR in NP specimens for S. pneumoniae diagnosis and standard microbiology as reference standard in patients with CAP, three studies confirmed the excellent NPA: two studies of patients in Western Europe with an NPA between 92 and 99% (17, 18) and a study performed in South Africa among HIV-infected adults, with an NPA at 92% (15).

In line with our results, a study comparing the performance of *lytA* in NP aspirate of patients admitted for CAP in Sweden found an increased PPA of the NP aspirate from 53% to 81% and a decreased NPA from 93% to 80% when lowering the detection cutoff value of the PCR (10^5^ to 10^2^ copies/mL) (18). However, the magnitude of the PPA increase in our study was lower, supporting the use of a cutoff at ≥10^4^ copies/mL in NP for S. pneumoniae diagnostic to keep a high NPA. (15–17). To our knowledge, our study is the first that evaluates different RT-PCR cutoffs in NP for H. influenzae.

Surprisingly, considering only the respiratory sample taken before antibiotics administration as the reference standard, we did not find a better NPA of FilmArray PP on NP. This can be explained by a good performance of standard microbiology, as only 12 (10%) patients received antibiotics more than 48 h before lower respiratory tract samples were collected.

Various retrospective studies suggested the utility of molecular diagnostic tests on LRT samples to deescalate or discontinue antibiotics (7–11). The high NPA of molecular tests on URT samples suggests the possibility to tailor antibiotic therapy in case of positive results, but the limited PPA does not support withdrawing antibiotics in case of negative results. In our population with CAP, we identified S. pneumoniae, H. influenzae, or M. catarrhalis in the NP swab of 38% of patients (45/118) who could have benefited from targeted antibiotics. However, because the first-line empirical antibiotic in patients with CAP is amoxicillin clavulanate, which is the treatment of choice of H. influenzae and M. catarrhalis, we expect an added value of a molecular test on the use of targeted antibiotics only in patients with S. pneumoniae (amoxicillin being the antibiotic of choice). However, only 9% (11/118) of patients had S. pneumoniae detected in the NP swab, limiting the potential impact of molecular tests in this CAP population.

Our study has several limitations. The first one, which is shared by all similar studies, is the absence of a gold standard for microbiological diagnostic in LRTIs, limiting the evaluation of new diagnostic tests. For this reason, we evaluated the performance of an NP swab molecular test using two reference standards, (i) standard microbiological tests (urinary antigen, LRT, and blood cultures), which are validated diagnostics tests (21), and (ii) the same molecular tests on LRT samples. This is important, as molecular tests have a higher diagnostic yield than standard microbiological tests, which may affect the NPA and falsely increase the discrepancy between culture and molecular tests (7, 8, 22, 23). Indeed, in our study, the NPA of the molecular analysis in NP swab was slightly lower using standard microbiological tests as reference standard than that using molecular analysis in LRT samples. Second, we used different PCR methods (FilmArray PP and in-house PCR) when analyzing the performance of a lower positive cutoff in NP. Nevertheless, comparing the performance of in-house PCR on NP and LRT shows similar results.

Third, our conclusions are limited to the three bacteria of interest, which are the main causes of bacterial community-acquired CAP (7). However, analyzing results of all bacteria included in the FilmArray Pneumonia plus panel (i.e., Gram-negative bacteria usually found in hospital-acquired infection) does not make sense in this population with community-acquired LRTI. Some studies also found a significant quantity of Staphylococcus aureus isolates and Gram-negative enteric bacilli in patients with CAP (24). However, these bacteria are described more frequently in severe pneumonia (25), in which withholding antibiotics is not an option.

Fourth, our small sample size and a selection bias of patients with good-quality sputa limit the generalizability of our conclusions. Indeed, we excluded 55% of patients because they did not reach the predefined criteria of sputum quality. As we tested a new diagnostic method, it was important to use the best possible reference standard. Using these inclusion criteria, 95% of patients received antibiotics, representing a population with LRTIs in which limitation of antibiotics is a challenge. However, we need more studies on this topic to evaluate the performance of other respiratory samples, such as low-quality sputa and oral and oropharyngeal swabs.

Finally, the optimal choice of URT sampling is still unknown, and we used only NP swabs. Previously cited studies comparing different URT and LRT samples showed an advantage of the NP swabs for identifying colonization by S. pneumoniae, no difference between NP and OP for H. influenzae, and an advantage of OP for M. catarrhalis (26). In our study, we chose the NP sample as they are already used in routine care for other molecular diagnoses.

Another limitation is that we used FilmArray PP, which is not validated for use in URT samples.

**Conclusion.** Our data showed that FilmArray PP in NP swabs for identifying the most common etiologies of community-acquired LRTI has a limited PPA, preventing its use for withholding antibiotics, but an excellent NPA, suggesting its use for targeted antibiotics in case of positive result, which is in line with the majority of previous studies. The real-life impact of rapid syndromic molecular testing should be tested in a randomized controlled trial.

## MATERIALS AND METHODS

### Study design and participants.

This is a retrospective study of adult patients (≥18 years) who attended the University Hospital of Lausanne, a tertiary care center in Switzerland, and who had available NP swab and LRT sample (sputa, lung aspirates, bronchoalveolar lavage) during two periods: February to May 2019 (phase 1) and November 2019 to August 2020 (phase 2). We chose these periods because of the systematic use of NP swab in patients with respiratory symptoms during the flu season and the COVID-19 pandemic.

Exclusion criteria were absence of LRTI (defined as an acute illness of ≤21 days, with cough as the main symptom, with ≥1 other lower respiratory tract symptom [sputum production, dyspnea, and/or chest discomfort/pain], and no alternative explanation) (27), presence of chronic lung disease (bronchiectasis, cystic fibrosis), hospitalization in an acute care hospital in the last 3 months, a low-quality sputum or lung aspirate (quality criteria defined as ≥25 neutrophils and <10 epithelial cells per low-power field) (28), upper and lower respiratory tract samples collected with a delay of ≥24 h, sample collection more than 48 h after admission, and refusal to participate in the study.

Patients’ demographics, comorbidities, symptoms, vital signs, radiology reports (chest X-rays or CT scans), and laboratory test results performed at admission were retrospectively recorded using a standardized electronic case report form in Research Electronic Data Capture (REDCap software). A radiologist reviewed chest X-rays that had no report available.

### Respiratory samples and microbiological analyses.

All respiratory samples were collected during routine care. Nurses performed the NP swabs (COPAN UTM with 3 mL of medium) according to standard institutional guidelines. If a patient had several LRT samples available, we chose first the bronchoalveolar lavage, then the lung aspirate, and finally the sputum. All respiratory samples were kept at −80°C within 24 h of collection. LRT samples were liquefied using a solution containing dithiothreitol to homogenize the specimens and improve the reliability of molecular tests, as described previously (29–31).

The NP swabs and LRT samples were tested with the FilmArray PP according to the manufacturer’s instructions. Results are expressed as semiquantitative results (<10^3.5^: negative; 10^4^, 10^5^, 10^6^, or ≥10^7^: positive) in DNA copies per mL for bacteria and as qualitative results (presence/absence) for atypical bacteria and viruses. We also tested all specimens with in-house real-time PCR on the automated molecular diagnostic platform of the Institute of Microbiology for (i) S. pneumoniae and (ii) H. influenzae using, respectively, *lytA* and *rdB* as targeted genes (15, 29).

### Statistical analyses.

We calculated the PPA, NPA, overall percent agreement (OPA) (32), positive and negative likelihood ratio, and positive and negative predictive values (PPV and NPV) of the FilmArray PP in NP swabs for the community-acquired respiratory bacteria of interest (S. pneumoniae, H. influenzae, M. catarrhalis) using two reference standards: (i) FilmArray PP in lower respiratory tract sample and (ii) a composite of standard microbiological tests (blood culture, LRT sample culture [considered positive if ≥10^5^ CFU/mL in sputum {7, 33}, ≥10^4^ CFU/mL in lung aspirates, or ≥ 10³ in bronchoalveolar lavage {34}], and S. pneumoniae urinary antigen).

We also calculated the performance of the FilmArray PP in NP swabs in a subgroup analysis: patients without prior use of antibiotics (to avoid the potential impact of antibiotics on cultures and a subsequent underevaluation of the specificity of the FilmArray PP in NP swabs).

Data were analyzed using RStudio software, version 1.3.1056 (Integrated Development Environment for R, RStudio, PBC, Boston, MA).

### Ethical approval.

All included patients signed a general consent form for research or provided written informed consent retrospectively. The study was accepted by the ethical commission of Canton de Vaud (Project-ID 2019-00961).
