# Supplementary material for: Utility of Polymerase Chain Reaction in Nasopharyngeal Swabs for Identifying Respiratory Bacteria Causing Community-Acquired Pneumonia
Source: Microbiol Spectr. 2022 May 18;10(3):e00379-22. doi: 10.1128/spectrum.00379-22 (PMC9241648; doi:10.1128/spectrum.00379-22)
Supplement: SUPPLEMENTAL FILE 1 — Supplemental material. Download spectrum.00379-22-s001.pdf, PDF file, 0.5 MB [file spectrum.00379-22-s001.pdf]

Supplementary Table 1: All microbiology results of participants (n = 118) by standard microbiology (SM) procedures (culture and PCR of any respiratory sample, hemoculture, urinary antigens) and *FilmArray® Pneumonia Panel plus* in the lower respiratory tract (FA-LRT) or nasopharyngeal (FA-NP) sample.

		SM ; n (%)	FA-LRT ; n (%)	FA-NP ; n (%)
	No pathogen detected	52 (44)	14 (12)	21 (18)
**Bacteria**				
	Any bacteria	46 (39)	90 (76)	73 (62)
	Haemophilus influenzae	19 (16)*	44 (37)	27 (23)
	Staphylococcus aureus	7 (5.9)*	29 (25)	28 (24)
	Streptococcus pneumoniae	12 (10)†	19 (16)	11 (9.3)
	Moraxella catarrhalis	0	14 (12)	9 (7.6)
	Escherichia coli	4 (3.4)*	11 (9.3)	2 (1.7)
	Streptococcus agalactiae	0	8 (6.8)	0
	Pseudomonas aeruginosa	3 (2.5)*	7 (5.9)	0
	Klebsiella pneumoniae	1 (0.8)*	5 (4.2)	1 (0.8)
	Enterobacter cloacae complex	0	4 (3.4)	1 (0.8)
	Mycoplasma pneumoniae	1 (0.8)‖	4 (3.4)	4 (3.4)
	Streptococcus pyogenes	2 (2.2)*	3 (2.5)	2 (1.7)
	Klebsiella oxytoca	1 (0.8)*	4 (3.4)	1 (0.8)
	Klebsiella aerogenes	1 (0.8)*	3 (2.5)	0
	Serratia marcescens	1 (0.8)*	3 (2.5)	0
	Proteus spp	0	1 (0.8)	2 (1.7)
	Chlamydia pneumoniae	1 (0.8)‖	1 (0.8)	1 (0.8)
	Legionella pneumophila	0	1 (0.8)	0
	Other bacteria ‡	6	-	-
**Viruses**				
	Any virus	28 (24)	67 (57)	57 (48)
	Human Rhinovirus Enterovirus	-	29 (25)	22 (19)
	Influenza A or B	12 (10)‖	17 (14)	12 (10)
	Coronavirus	-	9 (7.6)	7 (5.9)
	Respiratory Syncytial Virus	9 (7.6)‖	10 (8.5)	10 (8.5)
	Parainfluenza Virus	-	6 (5.1)	5 (4.2)
	Human Metapneumovirus	-	7 (5.9)	5 (4.2)
	Adenovirus	-	0	0
	SARS-CoV-2	2 (2.2)‖	-	-
**Antibiotics Resistance Gene**				
	CTXM	-	5 (4.2)	1 (0.8)
	mecAC and MREJ	-	2 (1.7)	3 (2.5)
	MERS	-	0	0
	IMP	-	0	0

KPC	-	0	0
NDM	-	0	0
OXA 48 like	-	1 (0.8)	0
VIM	-	1 (0.8)	0

* diagnostic by LRT culture; † positive test : urine antigen (6), LRT culture (10), blood culture (2); ‖ diagnostic by positive PCR on naso-pharyngeal swab, ‡ Streptococcus gr. anginosus (4), Neisseria perflava (1), Staphylococcus epidermidis (1)

Supplementary Table 2: Performance of *In house* PCR in naso-pharyngeal (NP) swabs at different cut off (≥10^2 and ≥10^3) and *FilmArray® Pneumonia Panel plus* (FilmArray PP) in NP swabs for detection of *S. pneumoniae* and *H. influenzae* in adults with community-acquired respiratory tract infections (n=118 patients) compared to different reference standard : *FilmArray® Pneumonia Panel plus* on a good quality lower respiratory tract (LRT) sample with positive cut-off value >10^4 copy/ml, or with the standard microbiology results. Positive percent agreement (PPA), negative percent agreement (NPA), o**verall percent agreement** (OPA), positive likelihood ratio (LR+), negative likelihood ratio (LR-), positive predictive value (PPV), negative predictive value (NPV).

**Target organism**	Reference standard	Diagnostic test	NP sample +	Reference standard +	PPA	NPA	OPA	LR +	LR -	PPV	NPV
S. pneumoniae											
	LRT PCR *FilmArray* PP										
		NP PCR In-House ≥10^2	15	19	0.68	0.98	0.93	33.87	0.32	0.87	0.94
		NP PCR In-House ≥10^3	12	19	0.58	0.99	0.92	57.32	0.43	0.92	0.92
		NP PCR *FilmArray PP* ≥10^4	11	19	0.58	1	0.93	Infinite	0.42	1	0.93
	Standard microbiology										
		NP PCR In-House ≥10^2	15	12	0.67	0.93	0.91	10.1	0.36	0.53	0.96
		NP PCR In-House ≥10^3	12	12	0.58	0.95	0.92	12.37	0.44	0.58	0.95
		NP PCR *FilmArray PP* ≥10^4	11	12	0.58	0.96	0.92	15.46	0.43	0.64	0.95
H. influenzae											
	LRT PCR *FilmArray* PP[4]										
		NP PCR In-House ≥10^2	50	44	0.77	0.78	0.78	3.57	0.29	0.68	0.85
		NP PCR In-House ≥10^3	35	44	0.64	0.91	0.81	6.73	0.4	0.8	0.81
		NP PCR *FilmArray PP* ≥10^4	27	44	0.61	1	0.86	Infinite	0.39	1	0.81
	Standard microbiology										
		NP PCR In-House ≥10^2	50	19	0.89	0.67	0.70	2.68	0.16	0.34	0.97
		NP PCR In-House ≥10^3	35	19	0.79	0.8	0.80	3.91	0.26	0.43	0.95
		NP PCR *FilmArray* PP ≥10^4	27	19	0.74	0.87	0.85	5.61	0.3	0.52	0.95
